# Photographic database of the European cave salamanders, genus *Hydromantes*

**DOI:** 10.1038/s41597-020-0513-8

**Published:** 2020-06-05

**Authors:** Enrico Lunghi, Simone Giachello, Yahui Zhao, Claudia Corti, Gentile Francesco Ficetola, Raoul Manenti

**Affiliations:** 1 Key Laboratory of the Zoological Systematics and Evolution, Institute of Zoology, Chinese Academy of Sciences, Beijing, China; 2 Museo di Storia Naturale dell’Università degli Studi di Firenze, Museo “La Specola”, Firenze, Italy; 3 Dipartimento di Scienze e Politiche Ambientali, Università degli Studi di Milano, Milano, Italy; 4 Univ. Grenoble Alpes, CNRS, Univ. Savoie Mont Blanc, LECA, Laboratoire d’Ecologie Alpine, F-38000 Grenoble, France

**Keywords:** Herpetology, Ecology

## Abstract

European *Hydromantes* are a group of eight salamander species often occurring in subterranean habitats, which are a difficult environment to explore. All *Hydromantes* are strictly protected species and thus, low-impact methodologies to study these salamanders are strongly needed. Here we used a photographic technique to produce a large dataset of European *Hydromantes*, providing standardised pictures of 1,052 individuals belonging to the eight species, including hybrids as well. With our methodology we were able to reduce the handling time of individuals, and produce high quality pictures useful to investigate multiple life traits of these endangered species. Furthermore, the standardised photos provided here can be used for future comparisons of individuals from the surveyed populations.

## Abstract (summary)

**Table:** 

Measurement(s)	morphometric parameter • color • length • biological sex • age • color pattern • body size trait
Technology Type(s)	photography
Factor Type(s)	salamander species • site of data collection • geographic location • salamander populations
Sample Characteristic - Organism	Hydromantes strinatii • Hydromantes ambrosii • Hydromantes italicus • Hydromantes flavus • Hydromantes supramontis • Hydromantes imperialis • Hydromantes genei • Hydromantes sarrabusensis • Hydromantes
Sample Characteristic - Environment	forest ecosystem • cave • mine
Sample Characteristic - Location	Autonomous Region of Sardinia • Region of Liguria • Region of Tuscany • Ariege

Machine-accessible metadata file describing the reported data: 10.6084/m9.figshare.12287222

## Background & Summary

The European cave salamanders (genus *Hydromantes*; see^1^ for taxonomic discussion) are a group of eight amphibians species endemic to Italy and to a small part of south-eastern France^2^. Three species (*H. strinatii*, *H. ambrosii* and *H. italicus*) are distributed along the northern and central Apennine chain (*H. strinatii* being the only species present in France), whereas five (*H. flavus*, *H. supramontis*, *H. imperialis*, *H. genei* and *H. sarrabusensis*) are endemic to Sardinia island, where geomorphology represents the main drive of their allopatry^2,3^. Only two mainland species, *H. ambrosii* and *H. italicus*, come into contact naturally and hybrid populations occur in a small area^4^. *Hydromantes* salamanders often have epigean activity during cold and wet seasons, but exploit subterranean habitats (such as caves, mines, small cervices and springs) to avoid unfavourable climatic conditions (when too hot and/or dry)^5–7^. These salamanders are lungless and require a specific combination of relatively low temperature and high moisture to efficiently carry out their cutaneous respiration;^2,8^ these conditions are often found in subterranean habitats^9^, thus *Hydromantes* are able to maintain stable populations and even reproduce there^10,11^, moving outdoors mostly to reach areas with high prey abundance^12,13^.

During the last few decades several studies have shed light on some life history traits of *Hydromantes*, such as the reproductive behaviour, trophic niche and population dynamics^14–17^. However, to collect data on these species can be extremely complex. First, the subterranean habitats are not human-friendly; some can be explored only by skilled speleologists and, in any case, the constant low temperature and the air moisture close to saturation represent a challenge for researchers spending prolonged time there^18,19^. Second, all the *Hydromantes* are strictly protected by both national and international laws^20,21^ and thus, studies cannot be performed without the proper authorisations. These salamanders are sensitive to multiple treats such as climate change, habitat degradation and poaching^2,22^. Furthermore, *Hydromantes* are sensitive to the deadly chytrid fungus, *Batrachochytrium salamandrivorans*, thus manipulation must be limited and must adhere to strict protocols to avoid spreading pathogens^23^. Indeed, researchers are continuously developing and testing different methodologies allowing them to limit their impact on these animals, without reducing the quality of their scientific researches^24–26^. Here we used a non-invasive method to build a large dataset related to *Hydromantes*, which can be used to investigate some of their unexplored life traits avoiding manipulation. Using photography, we here provide a dataset covering all *Hydromantes* species occurring in Europe, which also represents one of the few photographic datasets available for the animal kingdom. Our dataset is not made up of simple pictures (e.g.^27,28^), but we have adopted an ad hoc methodology to obtain standardised photos that can be used for future comparisons and analysed with multiple software^29–31^. There is a growing demand for publication of standardised dataset^32–34^, and those relating to endangered or not yet assessed species have a particular value^35,36^; however, special care should be taken when publishing data on sensitive species^37^. This dataset represents a snapshot of multiple *Hydromantes* populations, providing information on the minimum population size^38^, the age of individuals and morphometry^39,40^, and the variability of dorsal colouration^41–43^. Digitisation is a practice adopted to make manuscripts stored in various collections easily accessible to the public^44–46^. More recently, this trend also considers animal species^31,47^, providing undeniable help for the entire scientific community, as well a window for the public to discover the wonders of nature. Therefore, with our dataset we will provide the first digital collection showing the morphological diversity of all European *Hydromantes* species.

## Methods

### Experimental design

**Fig. 1:**
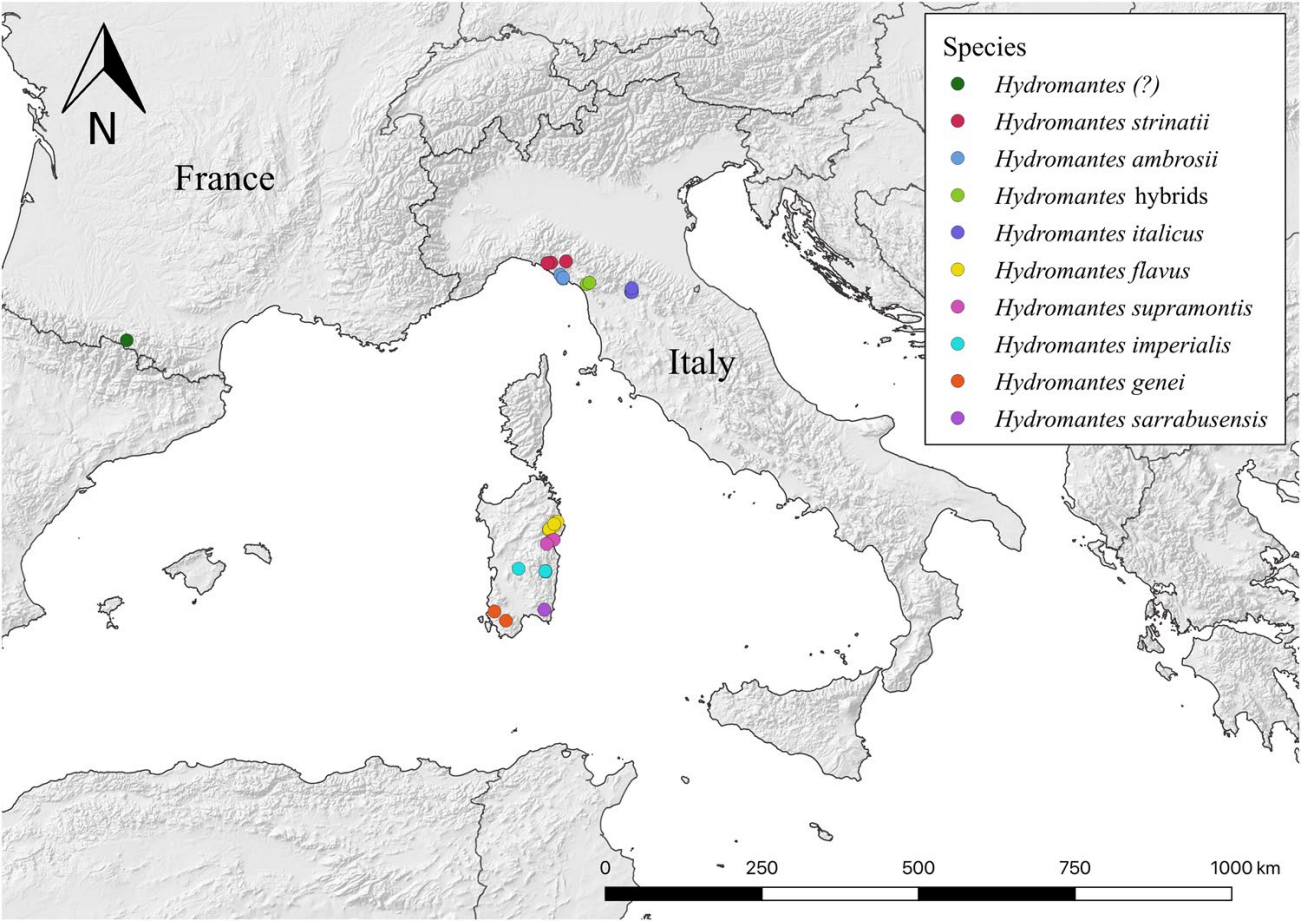
Map showing the locations where the *Hydromantes* salamanders were photographed.

We collected photographic information on all European *Hydromantes* species, also including hybrid populations. For each species and for the hybrid zone between *H. italicus* and *H. ambrosii*, we photographed ≥55 individuals, collecting data from at least two different sites (Table 1 and Fig. 1); this allowed us to gather information on a large number of individuals from different areas^2,4^. We also considered a population introduced into the French Pyrenees, which probably includes individuals of different *Hydromantes* species, if not hybrids;^48^ there, we photographed 47 individuals. The surveyed sites included forest, natural caves, mines and artificial springs (see Table 1); in all of them, the presence of *Hydromantes* was previously assessed^4,48,49^. Each site was surveyed once between August and October 2018 in order to avoid repeated photos on the same individual. Prior to each survey, equipment and shoes were cleaned and disinfected with bleach to avoid the spread of potential pathogens.

**Table 1:** Qualitative data of the *Hydromantes* photographic dataset^50^.

Column	Data description	Typology of data
1	ID	The salamander’s database code
2	Site	“Forest”, “Cave”, “Mine” or “Spring”
3–4	Latitude and Longitude	Low resolution coordinates of the site
5	Population	The population code
6–8	Country, Region and Province	The relevant information for each site
9–10	Month and Year	The date in which the picture was taken
11	Species	The species to which the individual belongs
12	N_photo	The unique file number corresponding to each individual
13	Age_class	Juvenile (0) or adult (1)
14	Sex	Adult males (M), adult females (F), juveniles (J)
15	Total_lenght	The total length of the individual (mm)
16	Eggs	Indicates if the female was gravid (1) or not (0). For males and juveniles (NA = not applicable)
17	Tail_issue	Indicates whether the tail is shown for its entire length (0) or not (1)
18	Scale_bar	Indicates the size of the picture scale bar (mm)

Information related to each photographed salamander and relative location. When the distance between two sites was <97 m, individuals were considered belonging to the same population^53^. Coordinates of the sites are not reported for species protection^37^.

### Individuals sampling

**Fig. 2:**
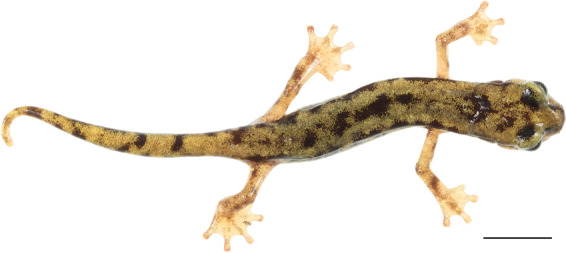
Example of *Hydromantes* picture from the database. Here an individual of *H. flavus* photographed using the described methodology; scale bar 10 mm.

In a dark area of the cave we built a photographic set to take standardised photos of salamanders. We used a soft white fabric box (hereafter, soft box) with a piece of white and flat plexiglass covering the inner bottom of the box. The soft box was placed suspended on the floor of the cave, with flash units on the left, on the right and under the soft box; such arrangement allows the light to be evenly distributed, limiting the presence of shadows on the subject (Fig. 2). The bottom flash produces a clear white background, a condition enabling a quick and easy way to isolate the salamander from the background in post-production. The two lateral flashes were arranged above the subject and inclined with 45° to fully and evenly illuminate it. The salamanders were collected and placed in fauna boxes until they were photographed. Before being photographed, each salamander was visually inspected and all debris adhering to the skin were removed. The salamanders were then placed in the soft box on the plexiglass and a photo was taken from above, keeping the camera perpendicular to the surface of the plexiglass. Salamanders were photographed with a Pantone colour card (see below) next to them to have a standard size reference and to correctly calibrate the colours and light during post-production. After the photo shoot, the salamanders were released where they were collected.

### Photo calibration

Correctly balancing the white is crucial to obtain standardised and comparable images. To guarantee an accurate and standardised white balance to our images, we shot a reference photo in RAW format (.CR2) of the Pantone colour card X-Rite Colorcheker Passport 2 at the beginning of each photographic session; shooting in RAW creates high quality files containing all the unprocessed data captured by the sensor. Images were then uploaded on a computer and organized in folders, each corresponding to the single photographic session. Using the function “White Balance Tool” of the software Adobe Camera Raw, for each site we created a custom white balance profile using the respective picture of the Pantone colour card as reference. We then applied the profiles to the respective images and converted them into JPEG format, which enables reduction in the size of pictures without compromising their quality.

## Data records

The dataset (The European *Hydromantes* salamanders^50^) includes 1,052 photographed individuals of all species and hybrids of European *Hydromantes*. Sites are within species natural range if not differently stated^2^. In particular, the dataset is composed as follows:

- *H. strinatii* (Strinati’s cave salamander): 82 individuals (40 females, 33 males, 9 juveniles) from five sites, two outside its natural range;
- *H. ambrosii* (Ambrosi’s cave salamander): 137 individuals (60 females, 47 males, 30 juveniles) from four sites;
- *H. italicus* (Italian cave salamander): 141 individuals (54 females, 53 males, 34 juveniles) from four sites;
- *H. flavus* (Monte Albo cave salamander): 171 individuals (64 females, 73 males, 34 juveniles) from six sites;
- *H. supramontis* (Supramonte cave salamander): 112 individuals (46 females, 25 males, 41 juveniles) from three sites;
- *H. imperialis* (Imperial cave salamander): 116 individuals (26 females, 63 males, 27 juveniles) from four sites;
- *H. genei* (Gene’s cave salamander): 122 individuals (43 females, 51 males, 28 juveniles) from two sites;
- *H. sarrabusensis* (Sette Fratelli cave salamander): 69 individuals (30 females, 27 males, 12 juveniles) from two sites;
- *Hydromantes* hybrids: 55 individuals (32 females, 14 males, 9 juveniles) from two sites within the hybrid zone^4^;
- *Hydromantes* (?) allochthonous population: 47 individuals (28 females, 11 males, 8 juveniles) from one site^48^.

Together with the photographic dataset we include information relating to each individual and their location (Qualitative data of the *Hydromantes* photographic dataset^50^; Table 1). Some information related to the salamanders (i.e., age, sex and total length) is critical to increase the quality of the dataset. Adult *Hydromantes* can be sexed with high confidence only by checking the presence of the mental gland on the chin of sexually mature males;^2^ this is a part of the body not visible from our images. With this additional information, salamanders can be divided into juveniles, adult males and adult females.

## Technical Validation

This dataset shows a unique collection of multiple individuals belonging to all *Hydromantes* species present in Europe. Collecting data on these salamanders can be challenging, as all species are strictly protected^20^, and even simple manipulation requires ministerial authorisations. The single survey performed on each site provides data on 1,052 different individuals. The methodology applied here enables production of standardised high quality images with low impact on the species^29,30^. The overall time required to shoot each salamander was usually <15 seconds, thus limiting the stress caused by handling^51^. The white calibration before each session avoided potential divergence in light condition and thus, providing standardised pictures. Our methodology allowed to avoid the hurdles due to the use of flash on animals with moist skin, being thus widely applicable. Blind measurements of salamanders were performed to reduce possible bias^52^. Salamanders were measured entirely or at the furthest visible point. In few cases, the tail was severed or its tip covered; these cases are indicated in Table 1^50^. Possible outliers were identified by plotting the data; the measurement of related individuals was taken twice to check whether the abnormal value was due to measurement errors. Considering that in these species individuals are aged according to their body size, and adults are sexed basing on the presence/absence of the male mental gland^2^, we added such information for each individual in Table 1^50^. To identify juveniles, we used the size of the smallest male observed as reference. Mainland species, hybrids and *H. genei* have all comparable size^35^, therefore we used 68 mm as threshold for these salamanders. The other four Sardinian species are defined as “giant”; for these species the size of the smallest male was 77 mm. All salamanders smaller than the respective reference male were considered juveniles. *Hydromantes* can live more than 10 years^2,53^ and thus, considering that our dataset provides a snapshot of individuals from 2018, it can be employed in comparative studies for multiple years ahead. The morphometrics obtained from our dataset can be compared with those of the same species published >20 years ago^54^ to assess whether any potential change occurred and which may be the cause^55,56^.

## Usage Notes

The pictures can be used in R environment (http://www.R-project.org/) to perform analyses on colouration (e.g.^57^) and geometric morphometrics (e.g.^58^), and with the program ImageJ to record multiple salamanders’ morphometrics (e.g.^59^). Furthermore, considering that, at least, in adult *Hydromantes* the dorsal pattern does not change throughout time^60^, this dataset can be used over time as a reference for the recognition of individuals belonging to the same population^61,62^, allowing to study growth rate, home range and other life traits. This also limits the manipulation of individuals, which represents a potential source of both stress and pathogens^23,51,63,64^.

## Data Availability

No code was used in this study.
